# Perioperative Management of a Donation After Circulatory Death Heart Transplant in a Recipient With a Persistent Left Superior Vena Cava

**DOI:** 10.7759/cureus.48459

**Published:** 2023-11-07

**Authors:** Sloan Long, Cole Sorrels, Saravanan Ramamoorthy, Juan C MacHannaford

**Affiliations:** 1 College of Medicine, Texas A&M College of Medicine, Dallas, USA; 2 Anesthesiology and Perioperative Medicine, US Anesthesia Partners, Dallas, USA; 3 Anesthesiology, Baylor University Medical Center, Dallas, USA; 4 Anesthesiology, Medical City Healthcare, Dallas, USA

**Keywords:** paroxsymal atrial fibrillation, nonischemic cardiomyopathy, heart failure with reduced ejection fraction, prosthetic graft, orthotopic heart transplant, persistent left superior vena cava (plsvc), donation after circulatory death (dcd), implantable cardioverter-defibrillator (icd)

## Abstract

A 60-year-old male with end-stage heart failure due to non-ischemic cardiomyopathy and mitral regurgitation presented for a donation after circulatory death (DCD) orthotopic heart transplantation. Intraoperatively, a persistent left superior vena cava (PLSVC), absent innominate vein, and small right superior vena cava were discovered. A bicaval technique was performed, using an interconnecting prosthetic conduit to anastomose the PLSVC with the right atrial appendage and an interposition graft to the native R SVC. After surgery, a transthoracic echocardiogram showed a left ventricular ejection fraction of 60-65% and improved systolic function. The postoperative course was uneventful, with discharge home after 16 days.

## Introduction

Persistent left superior vena cava (PLSVC) is a common congenital venous abnormality, with an overall prevalence of 0.3% to 0.5% [1-6]. Prevalence in patients with congenital heart disease is 2.8% to 4.3% [1-5]. Specific evidence can suggest the presence of a PLSVC prior to surgery, such as an abnormally positioned central venous catheter, mediastinal widening, or retrocardial echographic free space indicating a dilated coronary sinus [3-4]. In this case, a large PLSVC, absent innominate vein, and small right SVC were found intraoperatively at the beginning of surgical transplantation. This report highlights a case with unique surgical and anesthetic management techniques that were necessary to ensure a favorable outcome for a heart transplant recipient with a PLSVC found intraoperatively.

## Case presentation

A 60-year-old male with end-stage heart failure secondary to non-ischemic cardiomyopathy presented for an orthotopic heart transplant due to ventricular dysfunction. A previous echocardiogram revealed severe mitral regurgitation with a left ventricular ejection fraction of 15-20%, diffuse hypokinesis, and a massively dilated left atrium. In addition, there was no evidence of structural abnormalities seen on the chest X-ray. Other pertinent history includes a right-sided automated implantable cardioverter defibrillator (AICD), paroxysmal atrial fibrillation, nonsustained ventricular tachycardia, and previous ischemic stroke. Eventually, a suitable donor heart was found, and the patient was admitted for surgery. 

**Figure 1:**
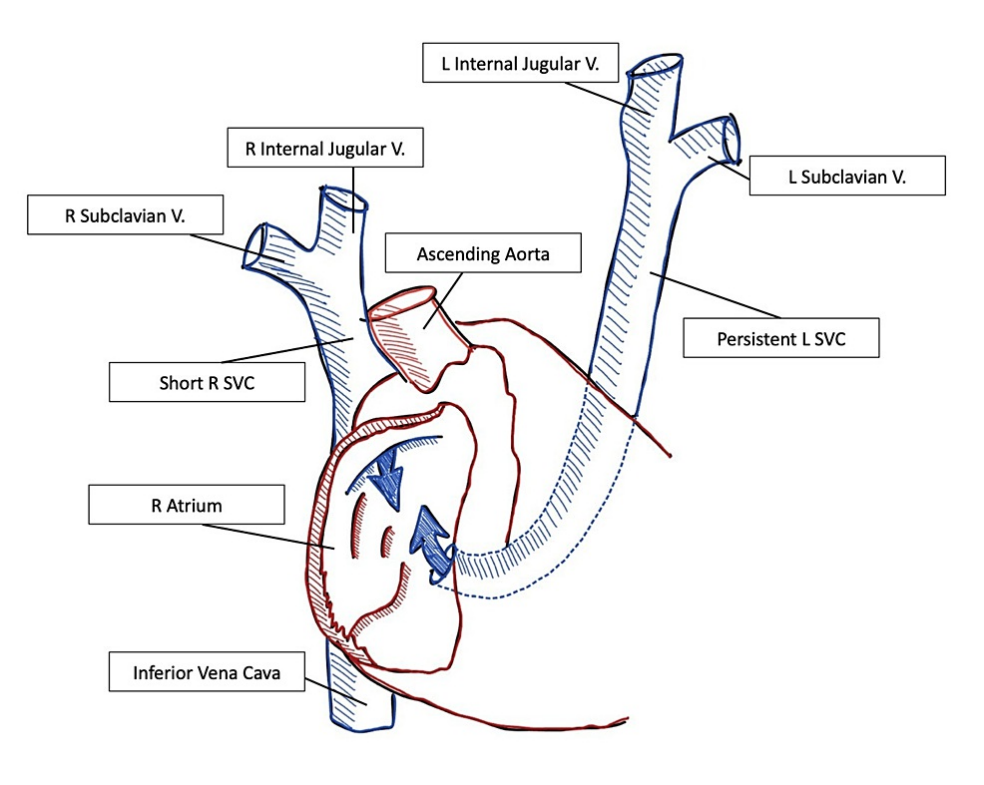
This figure demonstrates the recipient patient’s native anatomy that was discovered intraoperatively Image Credits: Cole Sorrels

The patient was brought to the operating room and an awake left brachial line was placed for arterial pressure monitoring. The patient was then endotracheally intubated after induction of general anesthesia. Based on the ultrasound assessment, the right internal jugular vein was observed to be too small for a central line placement. A 9 French, double-lumen multiple-access catheter (MAC) was placed in the left internal jugular vein with ultrasound and transesophageal echocardiogram (TEE) guidance. A 7 French triple-lumen catheter was placed in the left subclavian vein for infusion of medications. A Swan-Ganz catheter was positioned in the pulmonary artery using TEE guidance. A midline sternotomy was achieved after appropriate conditioning. After pericardiectomy, the recipient was found to have a large persistent left SVC, a small right SVC, and an absent innominate vein. This anatomy is depicted in Figure 1.

**Figure 2:**
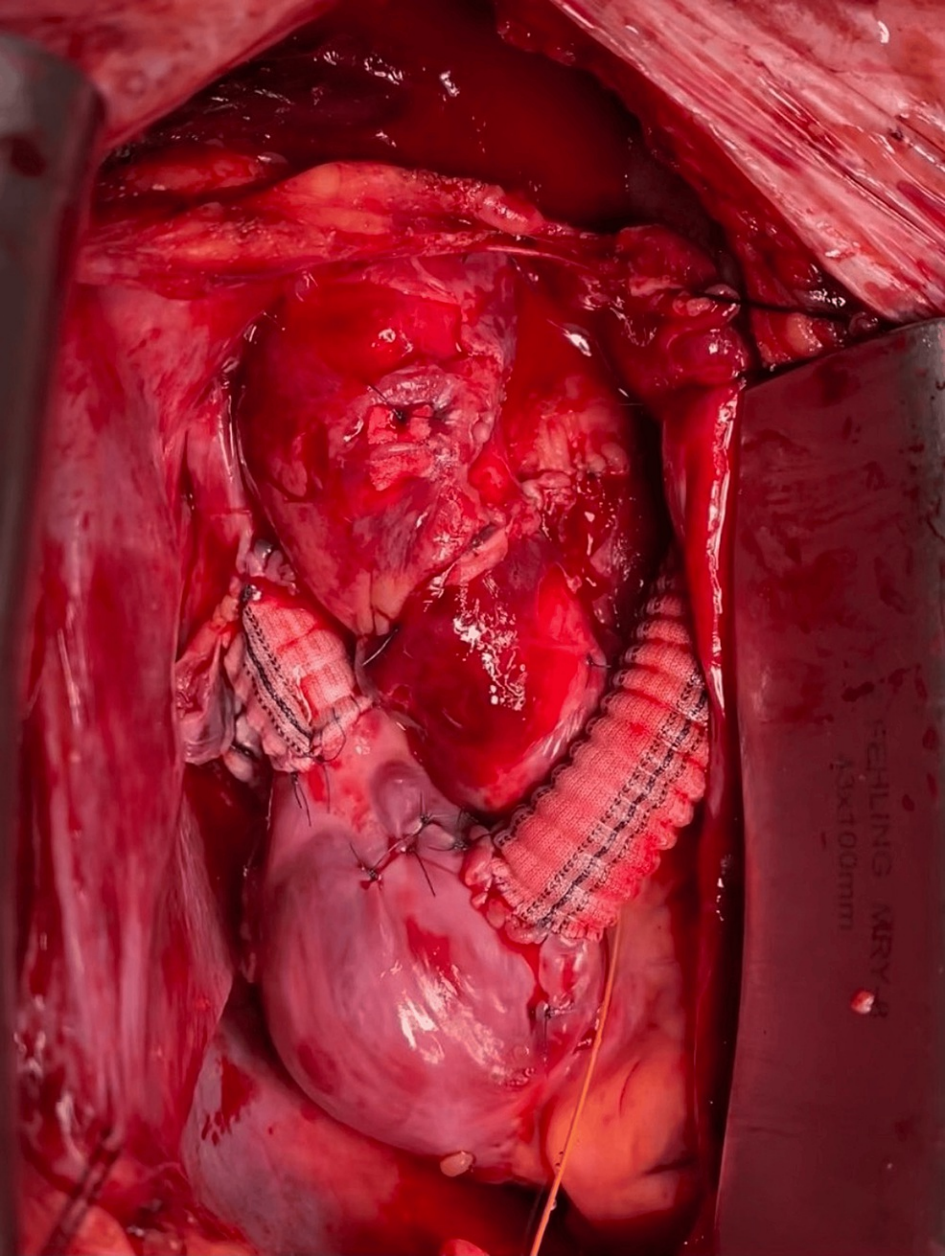
Post-heart transplant with a size 16 aortic prosthetic graft and a size 14 interposition graft to accommodate the patient’s anatomy

When the donation after circulatory death (DCD) heart arrived, the patient was heparinized, the IVC was cannulated for venous drainage, and the distal ascending aorta was cannulated for arterial flow. Cardiopulmonary bypass was established; however, upon further inspection, the stricture of the right SVC made it too small for bicaval cannulation, so the left SVC was drained via the MAC catheter placed in the left internal jugular vein. Caval tapes, a type of vascular tourniquet, were placed around the vena cava to block the flow of venous blood and air. Once appropriate activated clotting time was achieved, the patient was placed on full cardiopulmonary bypass without complication. While the aorta was cross-clamped and the native heart was excised, the left atrial anastomosis and donor pulmonary vessels were trimmed. Before connecting the DCD heart, a 16 mm aortic prosthetic graft was attached to the PLSVC followed by sequential anastomosis of the left atrium, IVC, pulmonary artery, and aorta. A cardioplegia solution was injected into the aortic root and between the anastomotic sites. The patient was placed in the Trendelenburg position, the heart was de-aired, terminal warm reperfusion (aka "hot shot") was given, and the aortic cross-clamp was removed. Given that the donor SVC was insufficient in length, a 14 mm interposition graft was added. The graft was anastomosed from the old PLSVC to the right atrial appendage; this is shown in Figure 2.

With ample biventricular function, the right AICD was removed, and the patient was weaned off cardiopulmonary bypass with only moderate inotropic support consisting of 0.03 mcg/kg/min of norepinephrine, 0.03 mcg/kg/min of epinephrine, and 5 mcg/kg/min of dobutamine. The patient was then decannulated, and protamine sulfate was given to achieve hemostasis. Bilateral mediastinal tubes were inserted, along with atrial and ventricular pacing wires.

Overall, the procedure was well-tolerated, and the postoperative recovery was smooth, with the exception of an episode of bacteremia despite the administration of routine antibiotics. However, this quickly resolved with a new antibiotic course. The patient did not experience any other complications and was discharged home after 16 days. The postoperative transthoracic echocardiogram showed improved cardiac function with normal systolic function and an estimated left ventricular ejection fraction of 60-65%. Left ventricular wall thickness and cavity size were also normal.

## Discussion

DCD heart transplants began in the early 2000s but were often associated with poor outcomes compared to donors fulfilling brainstem death criteria [7]. From 2014-2018 and 2015-2020, DCD heart transplant programs performed by Chew et al. and Messer et al., respectively, confirmed a future for DCD donor hearts with recipients having a greater than 91% one-year survival rate post-transplant [7]. Alterations in techniques for donor heart resuscitation allowed for increased viability, and DCD heart transplants are becoming more widely performed today [7].

While PLSVC is a common congenital venous abnormality, it only occurs in about 0.5% of the normal population [1-6]. Some evidence can suggest the presence of a PLSVC prior to surgery, such as mediastinal widening or an abnormally positioned CVC on chest X-ray; however, there was no indication of a PLSVC seen in this patient's preoperative workup [5,8-9]. Thus, these anatomical abnormalities were found intraoperatively after a DCD heart transplantation was planned. Surgery proceeded using a bicaval technique and a size 16 aortic graft to anastomose the PLSVC to the R atrial appendage [1,3,5-6]. Upon noting the patient’s small R SVC, a size 14 interposition graft was added to complete the anastomoses.

Other techniques can be performed in bicaval orthotopic transplants such as end-to-side anastomosis of the PLSVC and R SVC by passing the LSVC between the aorta and pulmonary arteries [1]. However, this poses an increased risk of compression between the vessels [2]. Another method is anastomosis of both SVCs to the innominate vein of the donor graft [1]. In this case, prosthetic conduits were used. While these have a risk of infection, kinking, and thrombo-occlusion, our patient did well on routine antibiotics and anticoagulants [1-2].

## Conclusions

This case demonstrates a successful DCD orthotopic heart transplant using a bicaval surgical technique in a patient with an intraoperatively found PLSVC. Our patient was discharged 16 days after surgery with normal systolic function and a left ventricular ejection fraction of 60-65%. The postoperative course was uneventful due to the surgical and anesthetic techniques noted in this case report.
